# Decoupling Internalization, Acidification and Phagosomal-Endosomal/lysosomal Fusion during Phagocytosis of InlA Coated Beads in Epithelial Cells

**DOI:** 10.1371/journal.pone.0006056

**Published:** 2009-06-26

**Authors:** Craig D. Blanchette, Youn-Hi Woo, Cynthia Thomas, Nan Shen, Todd A. Sulchek, Amy L. Hiddessen

**Affiliations:** Physical & Life Sciences, Lawrence Livermore National Laboratory, Livermore, California, United States of America; University of Birmingham, United Kingdom

## Abstract

**Background:**

Phagocytosis has been extensively examined in ‘professional’ phagocytic cells using pH sensitive dyes. However, in many of the previous studies, a separation between the end of internalization, beginning of acidification and completion of phagosomal-endosomal/lysosomal fusion was not clearly established. In addition, very little work has been done to systematically examine phagosomal maturation in ‘non-professional’ phagocytic cells. Therefore, in this study, we developed a simple method to measure and decouple particle internalization, phagosomal acidification and phagosomal-endosomal/lysosomal fusion in Madin-Darby Canine Kidney (MDCK) and Caco-2 epithelial cells.

**Methodology/Principal Findings:**

Our method was developed using a pathogen mimetic system consisting of polystyrene beads coated with Internalin A (InlA), a membrane surface protein from *Listeria monocytogenes* known to trigger receptor-mediated phagocytosis. We were able to independently measure the rates of internalization, phagosomal acidification and phagosomal-endosomal/lysosomal fusion in epithelial cells by combining the InlA-coated beads (InlA-beads) with antibody quenching, a pH sensitive dye and an endosomal/lysosomal dye. By performing these independent measurements under identical experimental conditions, we were able to decouple the three processes and establish time scales for each. In a separate set of experiments, we exploited the phagosomal acidification process to demonstrate an additional, *real-time* method for tracking bead binding, internalization and phagosomal acidification.

**Conclusions/Significance:**

Using this method, we found that the time scales for internalization, phagosomal acidification and phagosomal-endosomal/lysosomal fusion ranged from 23–32 min, 3–4 min and 74–120 min, respectively, for MDCK and Caco-2 epithelial cells. Both the static and real-time methods developed here are expected to be readily and broadly applicable, as they simply require fluorophore conjugation to a particle of interest, such as a pathogen or mimetic, in combination with common cell labeling dyes. As such, these methods hold promise for future measurements of receptor-mediated internalization in other cell systems, e.g. pathogen-host systems.

## Introduction

Phagocytosis is central to the degradation of foreign particles such as pathogens and, as such, is a vital process in host defense. During phagocytosis, cells ingest invading pathogens into plasma membrane-derived vacuoles, referred to as phagosomes. This process is often receptor-mediated, and ultimately results in internalization of the pathogen into a phagosome via a complex sequence of events involving receptor clustering, kinase activation, remodeling of the actin cytoskeleton and an increase of membrane traffic (see [1], [2], [3] for review). Following internalization, the phagosome is transformed into a phagolysosome through a progressive maturation process that is dependent on the sequential fusion of endosomes and lysosomes with the internalized phagosome (see [3], [4] for review). The phagolysosome is characterized as being acidic (below pH 5.5) and rich in hydrolytic enzymes. The low pH is believed to enhance host defenses by inhibiting microbial growth and enhancing the activity of degradative enzymes. Interestingly, the pH drop in phagosomes was identified over 60 years ago [5] but only in the past two decades was it shown that this pH drop is not dependent on phagosome-endosomal/lysosomal fusion, but rather is mediated by a plasma-membrane derived, vacuolar-type H-ATPase (or V-ATPase) active in the phagosomal membrane [6], [7], [8]. After acidification, phagosomes undergo fusion with late endosomes and/or lysosomes [9], [10]. Although the process of particle internalization and phagosomal maturation is central to host defense, certain pathogens have evolved to evade some or all of the steps in the phagocytic pathway to gain access to the cell interior. For example, *Legionella pneumophila* [11], *Taxoplasma gondii* [12] and *Histoplasma capsulatum* [13] prevent phagosomal acidification and *Mycobacterium tuberculosis* [14], *Listeria monocytogenes* [15], *Chlamydia psitacci* [16], *T. gondii* [17], *Legionella pneumophila* [18], and *Mycobacterium avium* [19] prevent phagosome-lysosome fusion. As a result, extensive research has been directed toward characterizing how such organisms subvert the host cell's primary defense mechanisms, including the process of phagosomal acidification.

One of the most widely used methods to study the early steps of phagosome acidification is the use of pH dependent fluorescent probes such as fluorescein isothiocyanate (FITC) [7], [8], [11], [20], [21], [22]. This method was first pioneered by Ohkuma and Poole to measure the pH of macrophage lysosomes [23]. This study and subsequent studies demonstrated that the excitation spectrum of fluorescein was strongly pH dependent between pH 4 and pH 7.4, and one could use standard calibration curves to readily determine the pH of internalized vesicles such as phagosomes, pinosomes, and lysosomes. This method has been widely used to specifically measure the rate of acidification during entry of foreign particles, particularly bacterial pathogens. In these studies, the rate of acidification varied from 5–30 minutes depending on the experimental set-up and bacterial-host system examined [6], [7], [8], [13], [19], [24]. As discussed above, prior to phagosomal maturation (phagosomal acidification followed by phagosomal-endosomal/lysosomal fusion), the pathogen/particle must be internalized into the cell. While the aforementioned studies have been vital in providing an initial understanding of phagosomal maturation (namely acidification), a separation between the end of internalization, beginning of acidification and subsequent phagosomal fusion to endosomes/lysosomes was not clearly established, and was often treated as a one-step process. More recently, a series of fluorescent fusion protein reporters were used to measure the onset of phagosomal maturation in living macrophages by monitoring co-localization of actin to the phagocytic cup, as well as distinct time scales for the progressive fusion of phagosomes to early and late endosomes/lysosomes [25]. This pH independent method was designed to quantitatively characterize the progression of vesicular fusion along the endocytic pathway in macrophages, and as such, measurements of phagosomal acidity were not incorporated. Since such studies are few, in general, more measurements are needed to kinetically characterize the step-wise process of phagocytosis, particularly for non-professional phagocytes where such data is lacking. For some cases, it would also be valuable to not only distinguish internalization from phagosomal maturation, but to further distinguish acidification from phagosome-endosome/lysosome fusion events, so as to provide a complete temporal characterization and permit direct comparison of key steps along the phagocytic pathway.

In this study, we built upon previous approaches to develop a method to decouple the kinetics of the step-wise process of internalization, phagosomal acidification and phagosomal-endosomal/lysosomal fusion during phagocytosis in non-professional phagocytic cells. To date, phagosomal acidification and phagosomal-endosomal/lysosomal fusion have been examined almost exclusively in ‘professional’ phagocytic cells, such as polymorphonuclear leukocytes (PMNs), monocytes, neutrophils and macrophages [26], [27] and, comparatively, very little work has been conducted to systematically examine this process in non-professional phagocytes, such as epithelial cells. Furthermore, it has been extensively documented that for professional phagocytes, phagosomal-endosomal/lysosomal fusion occurs after the phagosome has become acidified via a vacuolar-type H-ATPase. In contrast, such mechanistic data for ‘non-professional’ phagocytic cells is scarce in the literature, and the process of phagosomal maturation in these cells is not well understood. Apart from helping to fill the knowledge gap, it would be particularly beneficial to better understand the process in non-professional phagocytes since several pathogens are known to invade the human body through intestinal epithelial cells, including *Shigella*, *Yersinia*, *Salmonella*, and *Listeria monocytogenes* [28]. Thus, distinguishing the kinetics of internalization from those of phagosomal maturation (acidification and subsequent phagosomal fusion events), will provide details for the environmental time course encountered by pathogens that invade them, and should contribute more broadly to our knowledge of infectious disease.

In this study, we modeled the receptor-mediated phagocytosis of *Listeria monocytogenes* with a pathogen mimetic consisting of Internalin A coated polystyrene beads (InlA-beads), and employed this mimetic to measure internalization and subsequent steps of phagosomal acidification in Madin-Darby canine kidney cells (MDCK) or human intestinal Caco-2 epithelial cells, respectively. InlA is a protein expressed on the surface of *L. monocytogenes* and has been shown to promote bacterial internalization via receptor-mediated phagocytosis [29], [30], [31]. This process occurs through binding between InlA and E-cadherin, the latter a cell surface adhesion molecule normally involved in cell-cell adhesion and junction formation [32], [33], [34]. InlA-functionalized beads are effective mimetics of receptor-mediated phagocytosis because the protein is necessary and sufficient to promote internalization of both InlA-functionalized beads and *L. monocytogenes* in Caco-2 [32], [35] and MDCK cells [36]. We independently measured the rates of internalization, phagosomal acidification and phagosomal-endosomal/lysosomal fusion in epithelial cells by combining the InlA-beads with antibody sensitive (i.e. quenchable), pH sensitive and endosomal/lysosomal dyes, as follows: To determine the rate of internalization, we measured the fraction of InlA-beads, pre-labeled with Alexa488 pH independent dye, that was internalized at various time points through the addition of an Alexa488 quencher antibody. In a second set of independent measurements, we measured as a function of time the fraction of InlA-beads, pre-labeled with pH sensitive FITC dye, that were phagocytosed and underwent phagosomal acidification. Finally, in a third set of independent experiments, the fraction of unlabeled InlA-beads that were co-localized with an endosome/lysosome dye in cells pre-treated with LysoTracker Red was measured as a function of time to assess the kinetics of phagosomal-endosomal/lysosomal fusion. By independently measuring these three processes under identical experimental conditions, we were able to readily decouple internalization, phagosomal acidification and phagosomal-endosomal/lysosomal fusion by simply measuring the lag between the measured rate curves for these three processes. It is worth noting that the third step in this process, phagosomal-endosomal/lysosomal fusion, involves a series of vesicle fusion events with endosomes in different stages of maturation (i.e. early endosomes, late endosomes and lysosomes); however, in the development of our methods, we were first interested in measuring the average kinetics of all these different fusion events, so as to establish our approach for distinguishing the time scales of acidification from fusion. As such, a general endosomal/lysosomal dye was used in this study. However, the further decoupling of early endosomal from late endosomal/lysosomal fusion events, using our approach combined with more specific labels, is a subject of continuing work. Lastly, to develop an additional application of our technique, in a completely separate experiment we exploited the phagosomal acidification process to track, in *real-time*, single particle binding, internalization and phagosomal acidification in both MDCK and Caco-2 cells using FITC-labeled InlA-beads. We demonstrated the more general applicability of this method to dynamically track receptor-mediated phagocytosis by monitoring internalization of FITC-labeled fibronectin beads in NIH 3T3 fibroblast cells. Both the static and dynamic (real-time) methods developed in this study simply require the conjugation of a fluorophore to the foreign particle of interest in combination with the use of endosomal/lysosomal dyes; thus, these techniques should readily enable studies of the process of phagosomal acidification and maturation for a wide range of pathogen-host cell systems where uptake is initially receptor-mediated.

## Materials and Methods

### Materials

Minimum Essential Media (MEM) alpha and Dulbecco's modified Eagles medium (DMEM) high glucose was purchased from Invitrogen (Carlsbad, CA). Fetal bovine serum (FBS) and fetal calf serum (FCS) were obtained from Thermo Fisher Scientific (Waltham, MA). Madin-Darby canine kidney, Caco-2, and NIH 3T3 fibroblast cell lines were purchased from American Type Culture Collection (ATCC) (Manassas, VA). 2 µm carboxyl functionalized polystyrene microspheres were obtained from Bangs Laboratories (Fishers, IN). 1-ethyl–3-(3-dimethylaminopropyl) carbodiimide hydrochloride (EDC) and *N*-hydroxysulfosuccinimide (Sulfo-NHS) were purchased from Pierce (Rockford, IL). Fluorescein-5-isothiocyanate (FITC ‘Isomer I’), Alexa488, anti-Alexa488 quencher antibodies and LysoTracker Red DND-99 were obtained from Invitrogen. Fibronectin was purchased from BD Biosciences (Franklin Lakes, NJ). Restriction enzymes were purchased from New England BioLabs (Ipswich, MA) and the cloning vectors were purchased from commercial sources as indicated below. Anti-InlA antibody was kindly provided by Professor Pascale Cossart (Pasteur Institute, Paris). Genomic DNA was isolated from *Listeria monocytogenes* (ATCC, strain 19115) using standard microbiological procedures. All water used in these experiments was purified in a Milli-Q synthesis system (Millipore, Billerica, MA) with a resistivity ≥18.2 MΩ and pH 5.5.

### MDCK, Caco-2, and NIH 3T3 fibroblast cell culture

MDCK, Caco-2, and NIH 3T3 cells were grown in Minimum Essential Media (MEM) alpha with 10% fetal bovine serum (FBS), MEM alpha with 20% FBS and Dulbecco's modified Eagles medium (DMEM) high glucose with 10% fetal calf serum and 4 mM L-glutamine, respectively, in 5% CO_2_ to ∼passage 3 in a T-75 flask to confluency prior to storage. Aliquots of approximately 5×10^6^ cells were then frozen in liquid nitrogen in media containing 10% DMSO until further use.

### InlA cloning from *L. monocytogenes*

The extracellular domain of Internalin A (N-terminal 500 residues) was amplified from *L. monocytogenes* genomic DNA. The amplified product was ligated into a 2.1-TOPO^TM^ vector (Invitrogen, Carlsbad, CA) and transformed into *E. coli* DH5α. The amplified product was selected for the insert that was in the appropriate direction and digested with EcoRI and BamHI before ligation into a GST expressing vector, pGEX-6p-1 (GE Healthcare, Piscataway, NJ). The final construct pGEX-6p-1-InlA was transformed into *E. coli* DH5αand the final sequence was confirmed.

### InlA expression and purification

*L. monocytogenes* outer membrane protein, Internalin A (InlA), was expressed in an *E. coli* system following standard molecular biology techniques. The final construct pGEX-6p-1-InlA was re-transformed into *E. coli* BL21. The bacteria were grown to an OD_600_ of 0.6 at 37°C and then induced with IPTG (1 mM) for 4 hrs at 37°C. After stopping the growth on ice, the cells were spun down and the pellet was homogenized via sonication (Ultrasonic processor XL™, Farmingdale, NY). The desired InlA was overexpressed as a soluble protein with a molecular weight of 75 kDa including the GST probe. The supernatant was purified using glutathione-agarose beads (Pierce, Rockford, IL) as the manufacturer recommended. Briefly, the supernatant of homogenized cells was incubated with glutathione-agarose beads at room temperature for 30 min. The protein bound beads were washed several times before incubating with PreScission™ Protease (GE Healthcare, Piscataway, NJ) at 4°C for 4 hrs, which eluted InlA with a mass of 50 kDa at a final yield of 5 mg/L of culture. The purified protein was identified as functional InlA by immunoblotting against anti-InlA antibody (see supplementary information Figure S1.)

### Conjugation of InlA to carboxyl terminated polystyrene beads and subsequent attachment of Alexa488 or FITC

50 µl of 2 µm carboxyl-terminated polystyrene beads from the stock solution (∼2×10^10^ beads/ml) was diluted to a final volume of 1 ml in Milli-Q water. The bead solution was centrifuged at 3600 rpm for 15 minutes and re-suspended in a 0.1 M MES 0.8% NaCl buffer (pH 4.7) to 1 ml. 1 mg of 1-ethyl-3-(3-dimethylaminopropyl) carbodiimide hydrochloride (EDC) and 1 mg of *N*-hydroxysulfosuccinimide (Sulfo-NHS) was then added to the bead solution and incubated at room temperature with a gentle mixing for 20 minutes. The bead solution was then centrifuged at 3600 rpm for 15 minutes and re-suspended in water. After a second centrifugation step (3600 rpm for 15 minutes), the bead solution was re-suspended in PBS (pH 7.4) with the addition of 100 µg of the corresponding protein (InlA, fibronectin, FITC-fibronectin). The protein bead solution was then incubated at room temperature in a tube rotator (Miltenyi Biotec) for 2 hours followed by three subsequent centrifugation (3600 rpm for 15 minutes) and re-suspension steps (PBS, pH 7.4) to remove unbound protein.

InlA-beads were labeled with Alexa488 as recommended by the manufacturer. Briefly, InlA-beads were centrifuged (3600 rpm for 15 minutes) and re-suspended in 90 µl PBS (pH 7.4). 10 µl of 1 M sodium bicarbonate and 50 µg of Alexa488 TFP ester were then added to the InlA-bead solution and incubated at room temperature in a tube rotator for 30 minutes. The Alexa488/InlA-bead solution was then subjected to three centrifugation (3600 rpm for 15 minutes) and re-suspension steps (PBS, pH 7.4) to remove any free Alexa488.

For subsequent attachment of FITC to InlA-beads, InlA-beads were centrifuged (3600 rpm for 15 minutes) and re-suspended in 1 ml of 100 mM sodium borate. 50 µg of FITC was then added to the InlA-bead solution and incubated at room temperature in a tube rotator for 2 hours. FITC/InlA-bead solution was then subjected to three centrifugation (3600 rpm for 15 minutes) and re-suspension steps (PBS, pH 7.4) to remove any free FITC.

### Conjugation of FITC-fibronectin to carboxyl terminated polystyrene beads

When using the same procedure of unlabeled protein–bead conjugation followed by fluorophore attachment for fibronectin coated beads, the fluorophore conjugation efficiency was very low and the beads were highly aggregated. To overcome this issue, we followed the above procedure with the one modification that fibronectin was first conjugated to FITC prior to bead conjugation. 200 µg of fibronectin was mixed with 50 µg of FITC in 100 mM sodium borate to a final volume of 100 µl and allowed to incubate at room temperature for 2 hours. The mixture containing FITC-fibronectin and unreacted FITC (i.e. still free in solution) was then used for conjugation to 2 µm carboxyl terminated polystyrene beads. The unreacted FITC was not removed from solution prior to conjugation since the EDC/NHS activated carboxyl beads can only be conjugated to the amines on fibronectin; thus, unreacted FITC would not interfere with conjugation of FITC-fibronectin. Unbound FITC-fibronectin, as well as any unreacted free FITC, was subsequently removed from the FITC-conjugated InlA-bead (FITC/InlA-bead) solution during the last three centrifugation (3600 rpm for 15 minutes) and re-suspension steps (PBS, pH 7.4) after bead conjugation.

### Determining bead internalization and phagosomal acidification with Alexa488 and FITC labeled InlA-beads, respectively

Cells were maintained in T-25 flasks. These experiments were conducted on cells that were grown to ∼50% confluency in 35 mm glass bottom petri dishes (MatTek cultureware, Ashland, MA).

Petri dishes containing the cells (MDCK and Caco-2) were first cooled to 4°C. After the cells were maintained at 4°C for 15 minutes, 100 µl of either the Alexa488/InlA-beads or FITC/InlA-beads suspension, which was at bead density of ∼1×10^9^ beads/ml, was deposited into the petri dish (this volume was selected because this amount of beads was sufficient to cover the entire area of the bottom of the petri dish) and the dishes were centrifuged at 3600 rpm for 3 minutes. Immediately following centrifugation, the cells were rinsed extensively with 4°C media to remove unbound beads. The rinsing procedure used was as follows: The media was aspirated off and 4 ml of fresh 4°C media was added. Using a pipette, 1 ml of the media was repetitively removed and subsequently ejected over the cells to facilitate removal of unbound beads. The procedure was repeated three times. The petri dishes were then placed in a 37 °C humidified incubator with 5% CO_2_, which was then defined as time 0 [7], [8], [37]. For the Alexa488/InlA-bead experiments, cells were removed from the incubator at different time points and immediately fixed in 4% formaldehyde. After the cells were fixed, 5 µg of the Alexa488 quencher antibody (final concentration 2 µg/ml) was added to the petri dish and allowed to incubate for 30 minutes. For the FITC/InlA-bead experiments this method could not be used because the pH within the phagosome equilibrated with the cytoplasm after cells were fixed. Therefore, for these experiments, the cells were removed from the incubator at different time points and immediately imaged for only 3 minutes.

Bright field and fluorescent images of the beads and cells were then acquired using an Axiovert 200M microscope (Zeiss, Minneapolis, MN) in the multiple channel mode that was equipped with a Photometric CoolSNAP HQ CCD camera (Photometrics, Tucson, AZ). The Alexa488/InlA beads were imaged using a 450–490 nm excitation filter and a 505 nm emission filter, and the FITC/InlA-beads were imaged using two different excitation filters, 470 nm and 430 nm, and a 505 nm emission filter for both excitation wavelengths. The average fluorescence across individual beads was determined using the AxioVision Rel. 4.6 software. Since the fluorescence intensity of individual beads stacked on top of one another could not be determined, these beads were excluded from analysis. The absolute intensity of the individual beads in the Alexa488/InlA experiments was used to ultimately determine the fraction of beads that were internalized as a function of time. As for the FITC/InlA-bead experiments, the ratio of emission at excitation 470 nm and 430 nm was used to assess the pH of the environment in which the beads resided, *i.e*. in acidified phagosomes or not. The resulting internalization (Alexa488/InlA beads) and phagosomal acidification (FITC/InlA beads) curves were fit with a sigmoid function using IGOR Pro 5.05A software. The free parameters used in the fit were t_1/2_, peak value of the curve (max), rate of increase (rate) and base.

### Determining phagosomal-endosomal/lysosomal fusion with InlA beads and LysoTracker labeled cells

LysoTracker is a fluorescent dye that has been extensively shown to partition to acidified compartments within a cell, e.g. lysosomes and endosomes [38], [39], [40], [41]. The dye is membrane permeable and when cells are incubated with the dye at concentrations in the range of 50–100 nM, the dye will localize to lysosomes and endosomes. Therefore, to label the lysosomes and endosomes of MDCK and Caco-2 cells, 50 nM LysoTracker Red DND-99 was incubated with plated cells for 2 hours at 37°C. After incubation, the cells were rinsed 3 times with fresh media and both bright field and fluorescent images were acquired using the same microscope set-up described above to ensure the lysosomes and endosomes were efficiently labeled (Figure S2). Cells were then rinsed with 4°C media and centrifuged with unlabeled InlA-beads as described above and placed back in the humidified incubator at 37°C and 5% CO_2_. Cells were removed from the incubator at different time points and immediately imaged for only 3 minutes to assess whether the LysoTracker dye was co-localized with the unlabeled InlA-beads, which would be indicative of phagosomal-endosomal/lysosomal fusion. Fluorescent images were acquired using a 510–560 excitation filter and 590 emission filter.

### Measuring single FITC/InlA-bead and FITC/fibronectin-bead intensity as a function of time to *dynamically* track internalization and phagosomal acidification *in real-time*

Prior to real-time tracking of bead internalization and subsequent phagosomal acidification, cells were treated as described above. Petri dishes containing the cells were cooled to 4°C. After the cells were maintained at 4°C for 15 minutes, 100 µl of the bead solution (InlA-beads for MDCK and Caco-2 and fibronectin-beads for NIH 3T3) was deposited into the petri dish and the dish was centrifuged at 3600 rpm for 3 minutes. Immediately following centrifugation, the cells were rinsed extensively with 4°C media, as described above, to remove unbound beads. The petri dish was then placed under the Axiovert 200M microscope on a temperature controlled heating stage (Harvard Apparatus, Holliston, MA) that was preheated to 37°C. The temperature of the 100× objective used for these experiments was maintained at 37°C with an objective heater controller (Bioptechs, Butler, PA). As the sample warmed to 37°C, which took approximately 1 min, cells containing bound beads were brought into focus. Once a cell containing bound beads was aligned in the focal plane a time series of bright field and fluorescent images were acquired in the multiple channel mode of the Axiovert 200M microscope. Fluorescent images were obtained using a 450–490 nm excitation filter and a 505 nm emission filter. All individual beads examined experienced an abrupt rapid drop in fluorescent bead intensity indicating that the bead had become internalized and contained within an acidified phagosome.

## Results and Discussion

### Characterization of fluorescently labeled InlA-beads: Alexa488 labeled-bead quenching and FITC labeled-bead pH calibration curves

**Figure 1:**
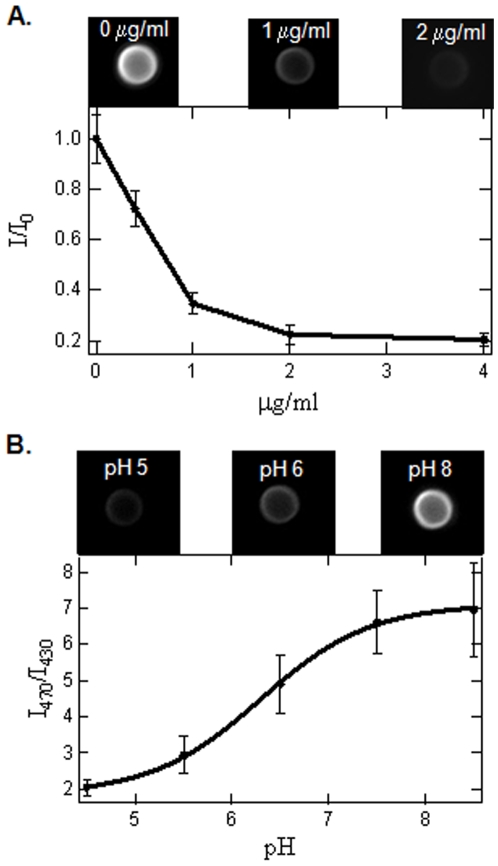
Dependence of Alexa488/InlA-bead fluorescence intensity before and after addition of quencher antibody and pH-dependence of FITC/InlA-bead fluorescence intensity. A.) Fluorescence intensity of InlA-Alexa488 beads as a function of the anti-Alexa488 concentration. B.) Ratio of the fluorescence intensities of FITC/InlA-beads at 430 and 470 nm excitation wavelengths across a range of pH of 4.5–8.5. A sigmoid relationship was used to calculate the pH after bead internalization. Bead size in both A. and B. is 2 µm.

It has been shown that binding of the anti-Alexa488 antibody to Alexa488 results in a fluorescence quench of Alexa488 [42]. Therefore, to determine the ability of the anti-Alexa488 antibody to quench the fluorescence of Alexa488 conjugated to InlA-beads, we incubated the anti-Alexa488 antibody at different concentrations with Alexa488/InlA beads for 30 minutes and analyzed the bead intensity as a function of anti-Alexa488 antibody concentration (Figure 1A). As can be seen in Figure 1A, an increase in the anti-Alexa488 concentration resulted in a decrease in the average Alexa488/InlA-bead intensity. This effect saturated at a concentration of 2 µg/ml, where no significant decreases in Alexa488/InlA-bead intensity was observed at higher anti-Alexa488 antibody concentrations. Therefore, we used this saturating concentration, 2 µg/ml, to assess bead internalization in the *in vitro* assays discussed below.

The excitation spectrum of FITC is highly sensitive to changes in pH [23]. The pH-dependent excitation properties of FITC have been typically measured using a ratiometric approach [7], [8], [20], [22], [23]. This method involves measuring the ratio of the fluorescence emission intensity when excited at two different wavelengths, where the two excitation wavelengths typically range from 450–490 nm and 400–450 nm. Using this ratiometric approach, it has been shown that the emission intensity at the higher excitation wavelength increases more at higher pH values than does the emission at the lower excitation wavelength. To determine if FITC conjugated to InlA-beads displayed this similar pH dependency, the 470/430 nm excitation ratio of FITC/InlA-beads, which is defined as the ratio of fluorescence intensity measured at a 505 nm emission wavelength when excited at 470 nm and 430 nm, were imaged in different pH adjusted media. Using this excitation ratio, FITC/InlA-beads were found to be highly pH-dependent between pH 4.5 and pH 8.5 (Figure 1B). As shown in Figure 1B, an increase in pH from pH 4.5 to pH 8.5 resulted in a corresponding 3.5 fold increase in the 470/430 excitation ratio of the FITC/InlA-beads. This change in excitation ratio was accompanied by a 10-fold increase in fluorescence intensity at 470 nm excitation and a 3-fold increase in fluorescence intensity at 430 nm excitation between pH 4.5 and pH 8.5. The increase in the 470/430 excitation ratio at higher pH values followed a sigmoid-like relationship, which has been observed in previous studies [6], [7], [13], [21], [23], [24], [43]. Therefore, the data was fit with a sigmoid function for subsequently determining the pH in phagosomes containing internalized FITC/InlA-beads.

### Independent measurements to decouple the steps of internalization, phagosomal acidification and phagosomal-endosomal/lysosomal fusion during phagocytosis of InlA-beads by epithelial cells

In three separate sets of experiments, we used Alexa488/InlA-beads, FITC/InlA-beads, and a combination of unlabeled InlA-beads with an endosomal/lysosomal dye, to independently measure the separate processes of internalization, phagosomal acidification and phagosomal endosomal/lysosomal fusion, respectively, during phagocytosis by MDCK and Caco-2 cells. The methods used in these experiments enabled us first to verify that each of these three distinct processes occurred and could be independently measured, and second, to establish a time-course or rate for each of these processes. In this way, we were able to distinguish the time-scales for internalization, phagosomal acidification and phagosomal-endosomal/lysosomal fusion, and provide a more accurate measure of the process and mechanism of acidification during phagocytosis by non-professional phagocytes.

**Figure 2:**
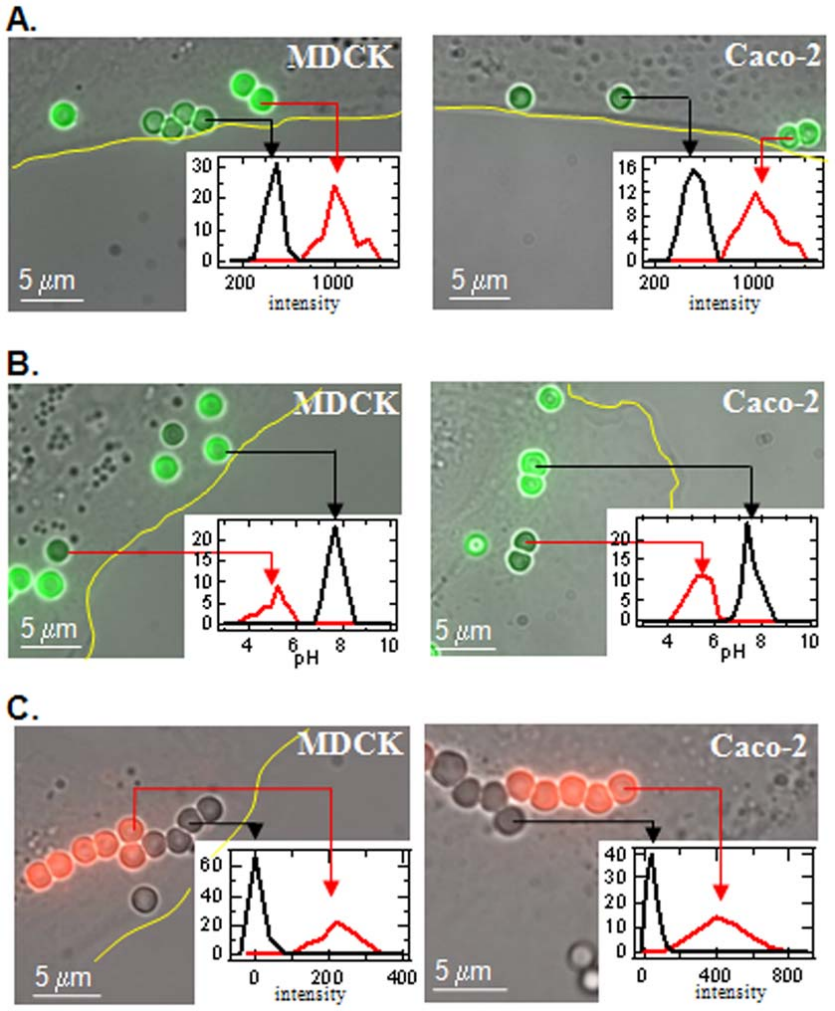
Distinguishing the processes of internalization, phagosomal acidification and phagosomal-endosomal/lysosomal fusion during phagocytosis in epithelial cells via independent, fluorescence-based measurements of Alexa488/InlA-beads, FITC/InlA-beads or unlabeled InlA-beads combined with a red lysosome/endosome dye, respectively. A.) MDCK cells (left panel) and Caco-2 cells after a 20 min or 30 min incubation period, respectively, with Alexa488/InlA-beads. Images were acquired after a subsequent, 30 min incubation period with the anti-Alexa488 quencher antibody. Insets – histogram of the fluorescent intensity of individual Alexa488/InlA-beads (bin width = 40 counts). The bimodal distribution results from bead internalization by the cell, where the higher fluorescence intensity peak (red line) represents internalized beads (inside the cell) and the lower intensity peak (black line) represents beads not internalized (outside the cell). B.) MDCK cells (left panel) and Caco-2 cells after a 20 min or 35 min incubation period, respectively, with FITC/InlA-beads. Insets – histogram of the pH of individual FITC/InlA-beads (bin width = 0.25). Bimodal distribution results from internalized beads that exist in acidified phagosomes, where FITC/InlA-beads with lower pH values (red line) indicate beads residing within acidified phagosomes. C.) Lysotracker Red-labeled MDCK cells and Caco-2 cells after a 95 min or 135 min incubation period with unlabeled InlA-beads, respectively. The lysosomes and endosomes of the cells were labeled with Lysotracker Red prior to bead binding. Inset - histogram of the red fluorescent intensity of Lysotracker Red dye co-localized with the unlabeled individual beads (bin width = 50 counts). The bimodal distribution results from the co-localization of the red fluorescent Lysotracker dye with the unlabeled beads, which indicates that phagosomal-endosomal/lysosomal fusion has occurred. In all images, the yellow lines denote the edge of the cell and the arrows are included to indicate which fluorescent intensity group (peak) the individual beads correspond to in the bimodal intensity distribution.

In the first of three independent measurements, we used Alexa488/InlA-beads to verify and measure InlA-bead internalization. The MDCK and Caco-2 cells incubated with Alexa488/InlA-beads (as described in the Materials and Methods section) were placed in a 5% CO_2_ incubator at 37°C for 20 and 30 min, respectively. Cells were then removed from the incubator and immediately fixed, followed by the addition of 5 µg of the anti-Alexa488 antibody (final concentration 2 µg/ml). After a 30 min incubation period with the anti-Alexa488 antibody, bright field and fluorescent images of the cells and beads were acquired (Figure 2A). The intensity of each individual bead associated with the cell was analyzed and used to construct a histogram of bead intensity. As shown in the insets of Figure 2A, the measured intensities of the beads were clearly bimodal for both MDCK and Caco-2 cells, with well-separated intensity peaks corresponding to beads that are either outside (peak centered at (∼450 counts) or inside (peaks centered at ∼1100 counts) the cell. Therefore, the two peaks in this bimodal distribution were used to determine the number of beads that were internalized (higher intensity peak, Figure 2A – red line) and the number of beads that were not internalized (lower intensity peak, Figure 2A – black line). Interestingly, based on the previously established Alexa488 fluorescence quenching curve we expected a five-fold decrease in fluorescence intensity of non-internalized beads relative to internalized beads at the anti-Alexa488 concentration used (2 µg/ml); however, we only observed a 2.5 fold decrease. We believe the discrepancy results from the fact that a large fraction of the beads' surface area was bound to the cell and it was thus not accessible to the anti-Alexa488 quencher, leading to sub-maximal quenching. Despite this, these results clearly indicate that this method can be accurately used not only to assess whether a bead has been internalized or not, but also to establish a rate of internalization by counting the number of beads that have been internalized at a given time (further details on the kinetics provided later in the Results).

In the second set of independent measurements, to determine whether or not the phagosomes containing internalized beads become acidified, FITC/InlA-beads were incubated with MDCK and Caco-2 cells using a similar experimental protocol described above for the Alexa488/InlA-bead internalization experiments (see Materials and Methods), with the exception being that the cells were imaged live so as not to affect the pH gradient that may exist across the phagosomal membrane. After a 20 and 35 min incubation period for MDCK and Caco-2 cells, respectively, cells were removed from the incubator and imaged. Since fixing the cells eliminated the proton gradient across the phagosome (data not shown), the cells were imaged for 3 minutes at room temperature in these experiments to slow the process of internalization and phagosomal acidification during imaging. Fluorescent images were acquired at an excitation wavelength of 430 nm and 470 nm with an emission wavelength of 505 nm. Figure 2B shows a combined bright field and fluorescence image at excitation 470 nm, which clearly indicates that a population of beads had a significantly lower 505 nm emission intensity, suggesting acidification of phagosomes containing internalized beads. To determine the pH environment of individual beads, the 470/430 nm excitation ratio was determined for individual beads and the sigmoid function in Figure 1B was used to calculate pH. Using this analysis, a bimodal distribution in pH was observed for both the MDCK and Caco-2 cells (Figure 2B – inset). The mean+/−standard deviation for the lower pH peak was centered at pH 5.0+/−0.5 and pH 5.3+/−0.5 (Figure 2B – red line) for MDCK and Caco-2 cells, respectively, whereas the higher pH peak was centered at pH 7.6+/−0.4 and pH 7.5+/−0.3 (Figure 2B – black line) for MDCK and Caco-2 cells, respectively. The appearance of this lower pH peak indicates that a portion of the beads have become internalized and exist in acidified phagosomes. These pH values correspond very closely to values that have been measured in acidified phagosomes of professional phagocytes (4.5–5.5), such as macrophages [7], [13], [15], [19], [21] and neutrophils [21].

It has been well documented for professional phagocytes that initial phagosomal acidification is mediated by plasma-membrane derived, vacuolar-type H-ATPases active in the phagosomal membrane [6], [7], [8]. To determine if the same mechanism of phagosomal acidification occurs in ‘non-professional’ phagocytes, MDCK and Caco-2 epithelial cells were pre-incubated with concanamycin A, a known inhibitor of the vacuolar-type H-ATPase [44], [45], [46]. Next, the treated cells were incubated with FITC/InlA-beads following the same protocol used above. After monitoring the fluorescence intensity of the FITC/InlA-beads for 6.5 hrs in both MDCK and Caco-2 cells, no change in FITC intensity was observed (Figure S3), which indicates that, similar to previously reported findings with professional phagocytes, initial phagosomal acidification occurs through the vacuolar-type H-ATPase.

Finally, in the third set of our three independent measurements, to determine whether the acidified phagosomes containing InlA-beads undergo phagosomal-endosomal/lysosomal fusion, and to assess the time required for this process (further details provided later in the Results), the lysosomes and endosomes of MDCK and Caco-2 cells were labeled with LysoTracker dye. LysoTracker is a fluorescent dye that has been extensively shown to partition to acidified compartments within a cell, e.g. lysosomes and endosomes [38], [39], [40], [41]. The dye is membrane permeable, and when cells are incubated with the dye at concentrations in the range of 50–100 nM, the dye will localize to lysosomes and endosomes. Therefore, the lysosomes and endosomes of MDCK and Caco-2 cells were labeled by incubating 50 nM LysoTracker Red DND-99 with plated cells for 2 hours at 37°C. After incubation, the cells were rinsed 3 times with fresh media and both bright field and fluorescent images were acquired using the same microscope set-up described above to verify that the lysosomes and endosomes were efficiently labeled (Figure S2). Cells were then rinsed with 4°C media and incubated with unlabeled InlA-beads using the same experimental protocol described previously for the FITC/InlA-bead experiments. After a 95 and 135 min incubation period for MDCK and Caco-2 cells, respectively, cells were removed from the incubator and bright field and fluorescent images were acquired for 3 min. The fluorescent images were acquired using the emission and excitation filter cubes specific for the red fluorescence of the LysoTracker Red dye. At these time points, a significant fraction of the unlabeled beads was spatially localized to the same regions as the fluorescent signal originating from the Lysotracker Red dye, indicating that extensive phagosomal-endosomal/lysosomal fusion occurred. To quantify the fraction of beads contained within phagosomes that had undergone phagosomal-endosomal/lysosomal fusion, the intensity of individual beads was analyzed and used to construct a histogram of bead intensity. As shown in the insets of Figure 2C, the fluorescent intensity of the beads was clearly bimodal for both MDCK and Caco-2 cells. Therefore, the two peaks in this bimodal distribution were used to determine the number of beads that were associated with phagosomes that had undergone phagosomal-endosomal/lysosomal fusion (higher intensity peak, Figure 2C – red line) and the fraction of beads that were not (lower intensity peak, Figure 2C – black line). It is worth noting that the LysoTracker Red does not distinguish between endosomes in different stages (i.e. early endosomes, late endosomes and lysosomes), so the method outlined above can only be applied to measure endosomal and/or lysosomal fusion in general. This dye was employed because we were interested in measuring the average kinetics of the process of endosomal/lysosomal fusion rather than fusion events with specific types of endosomes. Characterization of the time scales for phagosomal fusion with different staged endosomes is currently being investigated with specific endosomal markers. However, since both endosomal and lysosomal fusion occur subsequent to phagosomal acidification, the method presented here can still be used to clearly distinguish these two processes during phagosomal maturation.

### Kinetics of InlA bead internalization and phagosomal acidification

**Figure 3:**
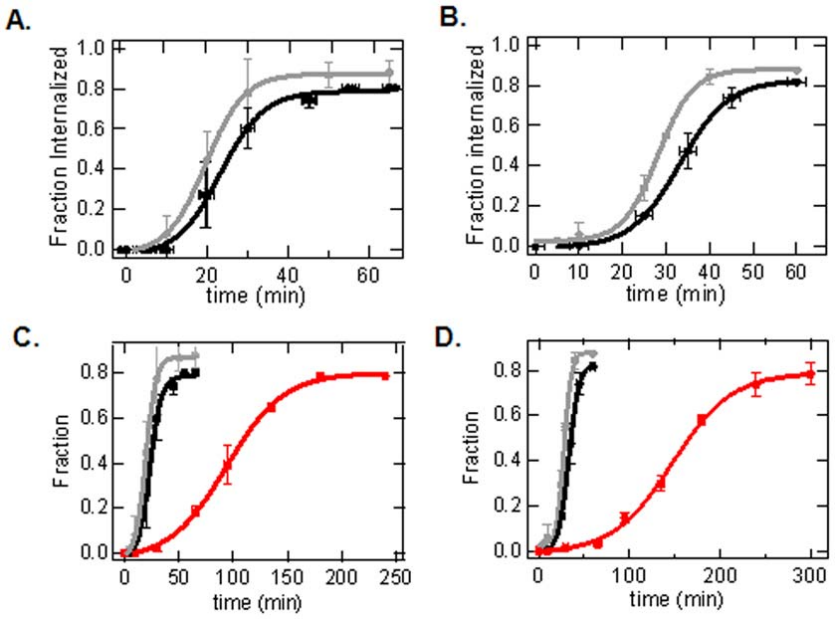
Rates of bead internalization, phagosomal acidification and phagosomal-endosomal/lysosomal during phagocytosis in epithelial cells via fluorescence-based measurements of Alexa488/InlA-beads, FITC/InlA-beads or unlabeled InlA-beads combined with a red endosome/lysosome dye, respectively, as a function of time. A.) Fraction of Alexa488/InlA-beads (gray line) internalized and fraction of FITC/InlA-beads (black line) residing within acidified phagosomes for MDCK cells as a function of time. B.) Fraction of Alexa488/InlA-beads (gray line) internalized and fraction of FITC/InlA-beads (black line) residing within acidified phagosomes for Caco-2 cells as a function of time. C.) Fraction of unlabeled InlA-beads co-localized with Lysotracker (indicative of endosomal/lysosomal -phagosomal fusion) for MDCK cells as function of time (red line). Internalization (Alexa488/InlA-beads) and acidification (FITC/InlA-beads) curves from A are also shown. D.) Fraction of unlabeled InlA beads co-localized with Lysotracker (indicative of phagosomal-endosomal/lysosomal fusion) for Caco-2 cells as function of time (red line). Internalization (Alexa488/InlA-beads) and acidification (FITC/InlA-beads) curves from B are also shown. Since the cells could not be fixed in the FITC-InlA-bead and Lysotracker Red+unlabeled InlA-beads experiments, images were acquired over a 3 min time period, which is the reason for the error bars on the time axis. In both cases, the FITC curve lags due to the time for phagosomal acidification. The Lysotracker curve significantly lags behind both the FITC curve and Alexa488 curve because the process of phagosomal-endosomal/lysosomal fusion occurs after bead internalization and phagosomal acidification.

Since the method described above allowed us to discriminate Alexa488/InlA-beads that had become internalized from those that had not, we further used it to calculate the rate of internalization by measuring the fraction of beads internalized at various time points after initial bead binding for MDCK and Caco-2 cells (Figure 3A and 3B – gray line). At each time point, >100 beads were analyzed. Using this method, we found that after ∼40 and ∼50 min, approximately 80% of the beads had become internalized for MDCK and Caco-2 cells, respectively. The fact that 100% bead internalization was not observed suggests that a fraction of the beads were non-specifically bound to the cell; thus, for these beads, the internalization pathway was most likely not activated. We used a sigmoid function to fit the data (as described in the Materials and Methods), and calculated the mean+/−standard deviation of the t_1/2_ for Alexa488/InlA-bead internalization to be 19.7+/−0.2 and 28.1+/−0.4 min for MDCK and Caco-2 cells, respectively (Table 1).

**Table 1:** Measured t_1/2_ (minutes) of InlA-bead internalization, phagosomal acidification and phagosomal-endosomal/lysosomal fusion in MDCK and Caco-2 cells.

Cell type	Internalization	Acidification	Endo-/lysosomal fusion
MDCK	19.7+/−0.2	3.5+/−0.8	74.3+/−1.9
Caco-2	28.1+/−0.4	4.0+/−0.4	119.5+/−2.8

To calculate phagosome acidification rates, the fraction of FITC/InlA -beads that were inside acidified phagosomes at various time points after initial bead binding for MDCK and Caco-2 cells was determined (Figure 3A and 3B – black line). This was done using the same experimental method described above, again with the exception being that at each time point cells were imaged live for 3 minutes rather than being fixed so as not to disrupt the natural acidification process. Again, at each time point >100 beads were analyzed. Using this method, we found that after ∼45 and ∼55 min the fraction of beads that existed in acidified phagosomes reached a maximum value of ∼0.80 for MDCK and Caco-2 cells, respectively. By fitting the data with a sigmoid function, the mean+/−standard deviation of the t_1/2_ for phagosomes containing FITC/InlA-beads to become internalized and acidified was calculated to be 23.2+/−0.8 and 32.1+/−0.2 min for MDCK and Caco-2, respectively

These combined results demonstrate that the FITC/InlA-beads curve lags behind the Alexa488/InlA-beads curve (Figure 3A and 3B). This can be attributed to the fact that the Alexa488/InlA curve is only a measure of the time it takes for bead internalization whereas the FITC/InlA-beads curve is a measure of the time it takes for a bead to become internalized and then for the phagosome to become acidified. In other words, the FITC/InlA-beads curve captures two steps, internalization and phagosomal acidification, whereas the Alexa488/InlA-bead curve captures only internalization. Since the only difference between these two curves is the time for phagosomal acidification, the difference in the t_1/2_ values from the Alexa488/InlA-bead curve and the FITC/InlA-bead curve can be used to calculate the rate of phagosomal acidification. Assuming two independent and non-overlapping steps, the mean+/−standard deviation for the rate of acidification was calculated to be 3.5+/−0.8 and 4+/−0.4 min for MDCK and Caco-2 cells, respectively (Table 1). These results indicate that, in these two non-professional phagocytic cell types, acidification is very rapid. It is worth noting that the InlA ligand used in this study simply turns on the internalization pathway via binding to E-cadherin at the cell surface, and thus should by itself not affect downstream processes such as acidification [33]. Therefore, we expect these results should hold for any phagocytic process for MDCK and Caco-2 cells where acidification is not modified by the internalized particles or pathogens. It is also worth noting that we are defining the rate of acidification as the time it takes for the phagosome to become completely acidified after the bead is completely internalized by the cell and the phagosome fully formed, and not the time when the vacuolar-type H-ATPase pumps are turned on. It is quite possible that the pumps are activated prior to the completion of bead internalization; however the phagosome itself cannot become acidified until bead internalization is completed and the phagosome is sealed, which is the parameter we aimed to measure in this study.

Phagosomal acidification rates have been reported to vary between 5–30 min for professional phagocytic cells. Examples of this include 10–15 min for phagocytosis of *Histoplasma capsulatum* by P388D1 macrophage-like cells [13], 10–20 min for phagocytosis of *Staphylococcus aureus* by thioglycolate-elicited peritoneal macrophages [7], 15 min for phagocytosis of *Mycobacterium avium* by J774 macrophages [19], 5–10 min for phagocytosis of sheep erythrocytes by P388D1 macrophage-like cells [8] and 10–30 min for phagocytosis of *Saccharomyces cerevisiae* by mouse peritoneal macrophages [24]. By comparison to the data obtained in this study, one may conclude that acidification is much more rapid in MDCK and Caco-2 cells than in professional phagocytes. However, in these previous studies, phagosomal acidification was not effectively decoupled from internalization; thus, acidification rates may have been overestimated. For example, in several of these studies, the fluorescently labeled pathogen was incubated with the cells at 4°C or room temperature for a given amount of time to allow for pathogen binding. This was followed by warming the cells to 37°C and then monitoring the fluorescence changes in the cell sample using a fluorescence spectrophotometer. Although this method has been shown to be very precise at capturing phagosomal acidification, it is much more limited in regards to accurately measuring acidification rates since internalization is not separated from acidification. For example, we have shown that in InlA-bead internalization and phagosomal acidification in MDCK and Caco-2 cells, the internalization step was the rate limiting step, whereas phagosomal acidification was quite rapid, and if FITC/InlA-beads were used alone as a measure of phagosomal acidification, the measured acidification rates would have been grossly overestimated. In addition, ensemble methods, such as fluorescence spectrophotometry, rely on averaging over a large population in which several phagosomes are in different stages of acidification; thus, only a gradual change in fluorescence is observed, and an abrupt change that may occur during a single phagosomal acidification event is not captured. This can result in significant broadening of the measured phagosome acidification rate. However, the method described here relies on independently measuring internalization and phagosomal acidification of internalized particles under identical experimental conditions; thus the process of internalization can be easily separated from phagosome acidification allowing for a more accurate measure of phagosome acidification rates.

### Kinetics of phagosomal-endosomal/lysosomal fusion

To assess the kinetics of phagosomal-endosomal/lysosomal fusion, an endosomal/lysosomal dye was used to determine the fraction of unlabeled InlA-beads existing in phagosomes that had undergone phagosomal-endosomal/lysosomal fusion as a function of time. As discussed above, LysoTracker Red was used to label lysosomes and endosomes, and co-localization of the LysoTracker Red dye with the unlabeled InlA-beads was used to quantify the fraction of beads that were contained within phagosomes that had fused with lysosomes and/or endosomes at different time points (Figure 3C and 3D – red line). This was done using the same experimental method described for measuring the kinetics of phagosomal acidification with FITC/InlA-beads, with the exception being that here the intensity of the Lysotracker Red dye co-localized with the bead inside the fused phagosome-endosome/lysosome was quantified; i.e. the beads were not labeled with a fluorophore so the appearance of the red fluorescent signal of the bead was due solely to the co-localization of the LysoTracker Red dye with the InlA beads. Using this method, we found that after ∼180 min and ∼210 min the fraction of unlabeled beads that were co-localized with the Lysotracker Red dye reached a maximum value of ∼0.80 for MDCK and Caco-2 cells, respectively. This maximal value corresponds very closely to the maximum value observed in the Alexa488/InlA-bead internalization assay and FITC/InlA-bead phagosomal acidification assay, which supports the notion that a small fraction (∼0.2) of the beads was non-specifically bound to the cell surface. After fitting the data with a sigmoid function, the mean+/−standard deviation of the t_1/2_ for phagosomal-endosomal/lysosomal fusion (including the time for bead internalization) was calculated to be 94.0+/−1.9 and 147.6+/−2.8 min for MDCK and Caco-2, respectively.

Based on these results, it appears that phagosomal-endosomal/lysosomal fusion occurs on a much slower time scale relative to phagosomal acidification (Figure 3C and 3D). Taking the difference between the t_1/2_ values calculated for bead internalization and phagosomal-endosomal/lysosomal fusion, phagosomal-endosomal/lysosomal fusion occurs 74.3+/−1.9 and 119.5+/−2.8 minutes after bead internalization in MDCK and Caco-2 cells, respectively (Table 1). This indicates that the process of phagosome-endosome/lysosome fusion is significantly longer than the process of acidification (3.5 and 4 min, respectively). Importantly, these findings for ‘non-professional’ phagocytic cells are consistent with what has been observed in professional phagocytes, that is, phagosomal acidification occurs prior to endosomal/lysosomal fusion [6], [7], [8], [9], [10]. However, the method of measuring the kinetics of phagosomal-endosomal/lysosomal fusion developed in this study was unique in that a direct comparison to phagosomal acidification rates could be made since both processes were independently measured after internalization. Although the reported kinetics of phagosomal maturation in professional phagocytes appear to vary depending on the particle and cell type employed, phagosomes typically begin to fuse with late endosomes 10–30 min after phagosomal formation [47], [48], [49], which is considerably faster than phagosomal-endosomal/lysosomal fusion rate measured here for MDCK and Caco-2 cells. Since phagocytosis is one of the main functions of professional phagocytes, it is not too surprising that phagosomal maturation is faster in these cell types than the non-professional phagocytes examined in this study.

### Tracking internalization and phagosome acidification of single beads in real-time using pH sensitive FITC labeled beads

Due to the rapid phagosomal acidification of internalized FITC/InlA-beads observed in the experiments described above, in a completely separate experiment, we examined the possibility of extending the same method to track, *in real-time*, the binding, internalization and phagosomal acidification of single FITC/InlA-beads. The aim of these experiments was to determine if we could exploit phagosomal acidification as a marker to track the pathway of a single bead dynamically, as opposed to the static measurements made earlier. Using this approach, we were able to track the path of single beads before and after internalization where the FITC/InlA-bead intensity drop was used as an accurate measure for phagosomal acidification. These experiments were initiated using a method that was similar to the previous experiments. FITC/InlA-beads were deposited into 35 mm glass petri dishes containing MDCK and Caco-2 cells and centrifuged at 3600 rpm for 3 minutes, while maintaining the cells at 4°C before and after centrifugation (refer to Materials and Methods). After centrifugation, cells were rinsed extensively with 4°C media to remove unbound beads and the petri dish was placed under an Axiovert 200M microscope on a temperature controlled heating stage that had been pre-heated to 37°C. As the sample warmed to 37°C, cells containing bound beads were brought into focus. Bright field and fluorescent images were then acquired at 3–5 min time intervals. To limit photobleaching, fluorescent images were acquired only using the 470 nm excitation and 505 nm emission filters.

**Figure 4:**
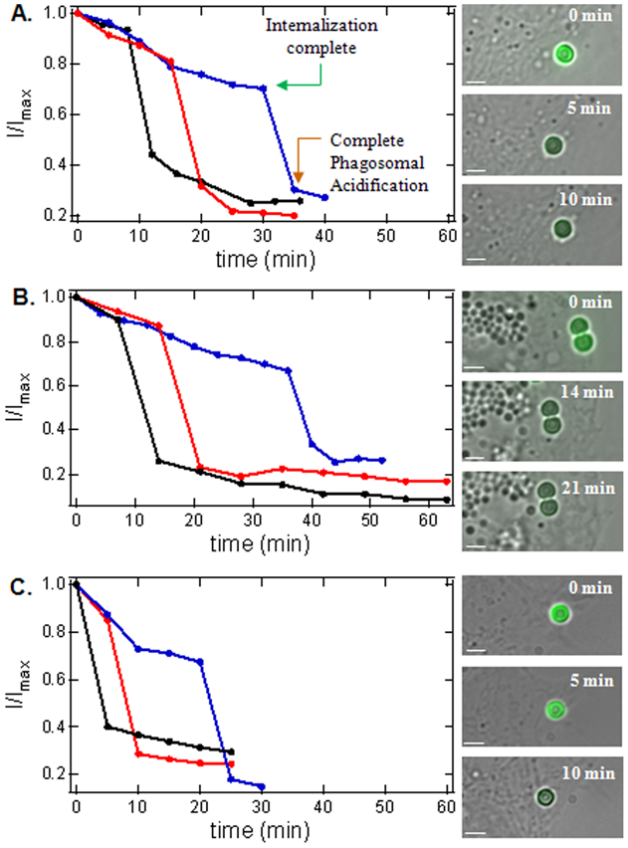
Application of FITC-labeled beads for *real-time* reporting of phagosomal acidification by tracking the fluorescence intensity of a single bead with time, from the time of initial binding to the cell through the completion of acidification, as demonstrated for FITC/InlA- in MDCK epithelial or Caco-2 epithelial cells or FITC/fibronectin-beads in NIH 3T3 fibroblast cells. A.) Real-time measurement of phagosomal acidification by tracking the fluorescence intensity of single FITC/InlA beads with time in MDCK cells. B.Real-time measurement of phagosomal acidification by tracking the fluorescence intensity of single FITC/InlA-beads in Caco-2 cells. C. Real-time measurement of phagosomal acidification by tracking the fluorescence intensity of single FITC/fibronectin-beads in 3T3 fibroblast cells. Images are representative of bead/cells before and after phagosomal acidification. Scale bar 2 µm (Three different lines, blue, red, and black, represent separate experiments with different bead).

When the intensity of an individual FITC/InlA-bead was monitored as a function of time, a sudden and rapid drop in FITC/InlA-bead intensity was consistently observed for both MDCK and Caco-2 cells (Figure 4A and 4B, respectively). The graphs in figures 4A and 4B show representative traces of the fluorescence intensity of 3 different FITC/InlA-beads as a function of time. This sudden drop in fluorescence intensity clearly indicates that the FITC/InlA-bead became internalized and resides in an acidified phagosome. The gradual decrease in intensity prior to the larger drop resulted from fluorescent photobleaching. Interestingly, this sudden drop in intensity occurs over a 3–5 minute time period, which corresponds very closely to the phagosomal acidification rates measured in the previous section. In addition, the time span for bead internalization and phagosomal acidification measured using this approach spanned the same time regime determined from the measurements in the previous section. Since this assay only reports the point of phagosomal acidification, we had no way of determining the exact point of bead internalization. However, the fact that the drop in fluorescence intensity occurred over a time period that corresponds closely to the acidification rates measured in previous experiments above suggests that the process of acidification is not only rapid but begins immediately after bead internalization is complete (where complete internalization is taken to be the time when the phagosomal membrane is fully sealed and distinct from the plasma membrane and extracellular space). Therefore, the initial point at which the FITC/InlA-bead intensity drops is expected to be a reasonably accurate measure, albeit less precise than the static bead measurements using Alexa488 quencher antibodies presented earlier, of the time when internalization is complete (Figure 4A – green arrow). Likewise, the time at which the sudden fluorescence intensity drop stops (or reaches the lower plateau) would be a reasonably accurate measure of the completion of phagosome acidification (Figure 4A – brown arrow). Based on this notion, one can use this technique to readily track the movement of a single pathogen or pathogen mimetic prior to internalization and the time course of acidification upon internalization (i.e. the time required for the fluorescence drop). The benefit of this method is that it only involves attaching a pH sensitive fluorophore, such as FITC, to the foreign particle (e.g. pathogen) of interest; thus, it could be used in a wide range of different pathogen and cell systems.

To demonstrate the range of applicability of this technique, we repeated these same experiments using a different protein and cell system; fibronectin and NIH 3T3 cells (see Supplemental Section S1 and Figure S4 for supporting information). In these experiments, the internalization of FITC/fibronectin beads was monitored in real-time in NIH 3T3 cells (Figure 4C) and we again observed a sudden drop (over ∼5 min) in the fluorescence intensity of individual FITC/fibronectin-beads (Figure 4C). These combined results suggest that this method holds promise to be used as an accurate real-time technique to dynamically track single particle phagocytosis and may be applicable to a wide range of pathogen-cell systems where uptake is initially receptor-mediated.

### Conclusions

In this study, we developed a simple method to decouple the processes of internalization and phagosomal maturation via three separate measurements, which allowed for a distinction between the time-courses for internalization and acidification, and significantly, provided distinct measurements and thereby comparison of the rates of phagosome acidification and phagosomal-endosomal/lysosomal fusion. This method was based on the use of anti-Alexa488 quenching of Alexa488/InlA-beads, pH sensitive FITC/InlA-beads, and a combination of unlabeled InlA-beads with cellular endosomal/lysosomal dye to independently measure internalization, phagosomal acidification and phagosomal-endosomal/lysosomal fusion, respectively. By independently measuring these three events under identical experimental conditions we were able to readily decouple the kinetics of both phagosomal acidification and phagosomal-endosomal/lysosomal fusion from bead internalization, a result which, to the best of our knowledge, has not been achieved previously for any class of phagocytic cells, professional or non-professional. Phagosomal acidification and endosomal/lysosomal fusion have been examined almost exclusively in ‘professional’ phagocytic cells, such as polymorphonuclear leukocytes **(** ***PMNs*** **)**, monocytes, neutrophils and macrophages [26], [27] and very little work has been conducted to examine these processes in non-professional phagocytes such as epithelial cells, despite the fact that several pathogens are known to invade the human body through intestinal epithelial cells, including *Shigella*, *Yersinia*, *Salmonella*, and *Listeria monocytogenes* [28]. Therefore, we applied our aforementioned method to measure phagosomal acidification and phagosomal-endosomal/lysosomal fusion in non-professional phagocytic cells, namely MDCK and Caco-2 epithelial cells, and found that phagosome acidification was very rapid, ranging from 3.5–4 min. Furthermore, we found that phagosomal-endosomal/lysosomal fusion was much slower, comparatively, ranging from 74–120 min. In addition to providing a detailed temporal characterization of phagocytosis and phagosomal maturation in non-professional phagocytes, the ability to measure and compare the kinetics of internalization from those of phagosomal maturation (acidification and subsequent phagosomal fusion events) should further contribute to understanding the interplay of host cell phagosomal acidification and maturation with the intracellular fate of invading pathogens, e.g. how a pathogen optimally orchestrates its escape from the phagosome to ensure its intracellular survival [50].

Finally, as an additional application of the pH sensitive FITC/InlA-beads developed in this work, we exploited the rapid phagosomal acidification process observed in the static measurements presented earlier, to track a single bead, *in real-time*, through binding, internalization and phagosomal acidification in both MDCK and Caco-2 cells. To demonstrate broader-range applicability, this method was verified by tracking real-time FITC-fibronectin/bead internalization and phagosomal acidification in 3T3 fibroblast cells. These results suggest that one can use this approach as a general method to *dynamically* track the path of individual beads, pathogens, etc. from the point of binding to the stage of phagosomal acidification through the simple conjugation of a pH sensitive probe such as FITC.

In conclusion, the methods presented in this study allow for independent measurements of, and thereby a decoupling of, the kinetics of three major processes involved in phagocytosis, namely internalization, acidification and endosomal/lysosomal fusion. In addition to using these static measurements to decouple the time-scales of these key steps, we extended the application of the pH sensitive FITC-labeled (conjugated to InlA or fibronectin-coated) beads to demonstrate an additional, broadly applicable method for the dynamic tracking of single beads as they bind, internalize and undergo acidification in phagosomes, as demonstrated for epithelial and fibroblast cells where phagocytosis is triggered by an initial cell surface receptor binding event.

## Supporting Information

Supplemental Section S1 Supporting information for Fibronectin-NIH 3T3 experiments and Concanamycin A experiments
(0.02 MB DOC)

Figure S1 (1.37 MB TIF)

Figure S2 (1.38 MB TIF)

Figure S3 (1.34 MB TIF)

Figure S4 (1.35 MB TIF)
