# Supplementary material for: Decoupling Internalization, Acidification and Phagosomal-Endosomal/lysosomal Fusion during Phagocytosis of InlA Coated Beads in Epithelial Cells
Source: PLoS One. 2009 Jun 26;4(6):e6056. doi: 10.1371/journal.pone.0006056 (PMC2699028; doi:10.1371/journal.pone.0006056)
Supplement: Supplemental Section S1 — Supporting information for Fibronectin-NIH 3T3 experiments and Concanamycin A experiments (0.02 MB DOC) [file pone.0006056.s001.doc]

**Supplemental**

Conjugation of Alexa488-fibronectin to carboxyl terminated polystyrene beads: For Alexa488 – fibronectin conjugation, 200 μg of fibronectin was mixed with 10 μl of 1 M sodium bicarbonate and 50 μg of Alexa488 TFP ester to a final volume of 100 μl and allowed to incubate at room temperature for 30 minutes. The mixed Alexa488-fibronectin and free Alexa488 solution was then used as the protein solution for conjugation to the 2 μm carboxyl terminated polystyrene beads using the same procedure described above. Free Alexa488 was subsequently removed from the Alexa488/InlA-bead solution during the last three centrifugation (3600 rpm for 15 minutes) and re-suspension steps (PBS, pH 7.4) after bead conjugation.

Internalization and phagosomal acidification of FITC/fibronectin-beads in NIH 3T3 fibroblasts: The internalization and phagosomal acidification of fibronectin beads in NIH 3T3 fibroblast cells were verified using Alexa488/fibronectin-beads and FITC/fibronectin-beads, as described for Alexa488/InlA-beads and FITC/InlA-beads in MDCK and Caco-2 cells. As was observed for those systems, FITC/fibronectin beads were internalized, and phagosomes containing internalized beads were acidified to pH ~ 5.5 (Figure S2). In addition, the rate of phagosome acidification for the FITC/fibronectin in NIH 3T3 fibroblast cells was measured to be 5.5 min.

Phagosomal acidification in the presence of Concanamycin A: To determine if phagosomal acidification in MDCK and Caco-2 epithelial cells occurs via the vacuolar-type H-ATPase, cells were pre-incubated with concanamycin A, a known inhibitor of the vacuolar-type H-ATPase [1,2,3]. The treated cells were then incubated with FITC/InlA-beads following the same protocol described in the Material and Methods section of the manuscript (Determining bead internalization and phagosomal acidification with Alexa488 and FITC labeled InlA-beads, respectively). The fluorescence intensity of the FITC/InlA-beads was monitored over 6.5 hrs in both MDCK and Caco-2 cells and the pH of the phagosome was quantified as described in the Material and Methods section of the text. After 6.5 hrs, no change in FITC intensity was observed (Figure S3), which indicates that, similar to previously reported findings with professional phagocytes, initial phagosomal acidification occurs through the vacuolar-type H-ATPase.

**Supplemental Figures**

**Figure S1.** Expression of functional Internalin A protein (InlA). A.) SDS-PAGE image (lane1: supernatant obtained from cell lysate; 2: marker in KDa: 3: flow through after binding expressed InlA-GST to glutathion-agarose beads: 4: wash: 5: Elution 1: 6: marker: 7: Elution 2. B.) Immunoblot image of purified InlA using anti-InlA antibody staining.

**Figure S2.** Labeling of lysosomes and endosomes with Lysotracker Red in preparation for measurements of phagosomal-lysosomal/endosomal fusion. Overlaid fluorescent/bright field image of MDCKs (A) and Caco-2 cells (B) after cells were incubated with Lysotracker for 2 hours. The bright red dots indicate that the lysosomes and endosomes were selectively labeled by the dye. Scale bar 10 μm

**Figure S3**. Phagosomal acidification does not occur in the presence of concanamycin A. MDCK cells (left panel) and Caco-2 cells, pretreated with concanamycin A, after a 420 min incubation period with FITC/InlA-beads. Insets – histogram of the pH of individual beads (bin width 0.25). The average pH of the phagosomes was 7.4 and 7.5 for MDCK and Caco-2 cells, respectively. The lack of a bimodal distribution in pH indicates that the phagosomes do not become acidified when the cells were pretreated with concanamycin A.

.

**Figure S4.** Measurements of bead internalization and acidification of phagosomes using fibronectin coated InlA beads and NIH 3T3 fibroblast cells. A.) Fluorescence/bright field overlay image of Alexa488/fibronectin-beads captured after 25.5 min of incubation with 3T3 cells, and a subsequent 30 min incubation with the anti-Alexa488 antibody quencher. Inset – histogram (bin width = 40 counts) of the fluorescent intensity of individual Alexa488/fibronectin-beads. The bimodal distribution results from bead internalization by the cell, where the higher fluorescence intensity peak (red line) represents internalized beads (inside the cell) and the lower intensity peak (black line) represents beads not internalized (outside the cell) B.) Fluorescence/bright field overlay image of FITC/fibronectin-beads after 25.5 min of incubation with 3T3 cells. . Inset – histogram of the pH of individual FITC/fibronectin-beads (bin width = 0.25). Bimodal distribution results from internalized beads that exist in acidified phagosomes, where FITC/fibronectin-beads with lower pH values (red line) indicate beads residing within acidified phagosomes. The yellow lines denote the edge of the cell and the arrows arrows are included to indicate which fluorescent intensity group (peak) the individual beads correspond to in the bimodal intensity distribution C. Fraction of Alexa488/fibronectin-beads (gray line) and FITC/fibronectin-beads (black line) internalized within 3T3 cells as a function of time. The FITC curve lags due to the time for phagosomal acidification.

1. Huss M, Ingenhorst G, Konig S, Gassel M, Drose S, et al. (2002) Concanamycin a, the specific inhibitor of V-ATPases, binds to the V-o subunit c. Journal of Biological Chemistry 277: 40544-40548.

2. Drose S, Bindseil KU, Bowman EJ, Siebers A, Zeeck A, et al. (1993) Inhibitory Effect of Modified Bafilomycins and Concanamycins on P-Type and V-Type Adenosine-Triphosphatases. Biochemistry 32: 3902-3906.

3. Drose S, Boddien C, Gassel M, Ingenhorst G, Zeeck A, et al. (2001) Semisynthetic derivatives of concanamycin A and C, as inhibitors of V- and P-type ATPases: Structure-activity investigations and developments of photoaffinity probes. Biochemistry 40: 2816-2825.
