# Supplementary figures and images for: Decoupling Internalization, Acidification and Phagosomal-Endosomal/lysosomal Fusion during Phagocytosis of InlA Coated Beads in Epithelial Cells
Source: PLoS One. 2009 Jun 26;4(6):e6056. doi: 10.1371/journal.pone.0006056 (PMC2699028; doi:10.1371/journal.pone.0006056)

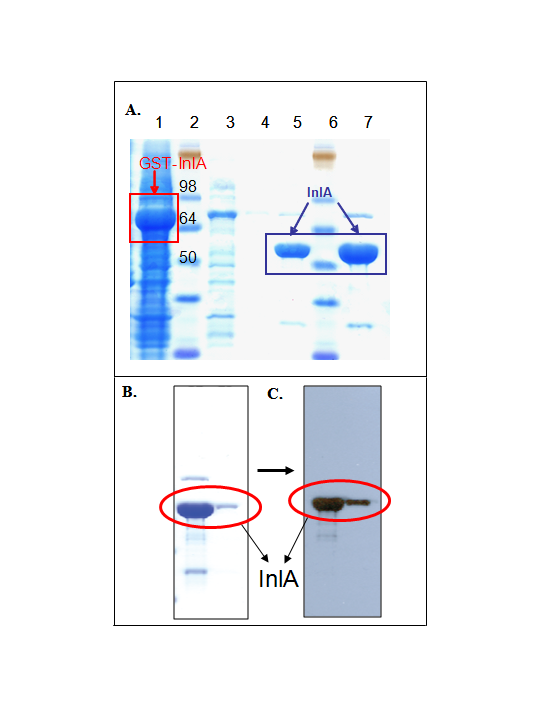

Supplement: Figure S1 — (1.37 MB TIF) [file pone.0006056.s002.tif]

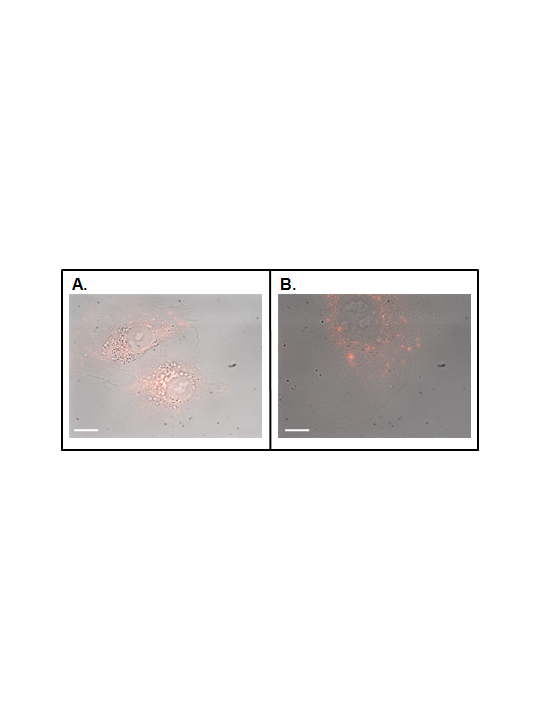

Supplement: Figure S2 — (1.38 MB TIF) [file pone.0006056.s003.tif]

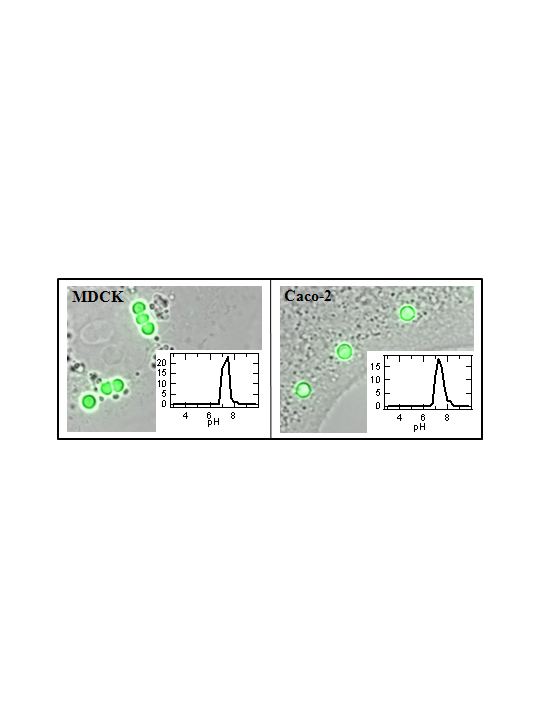

Supplement: Figure S3 — (1.34 MB TIF) [file pone.0006056.s004.tif]

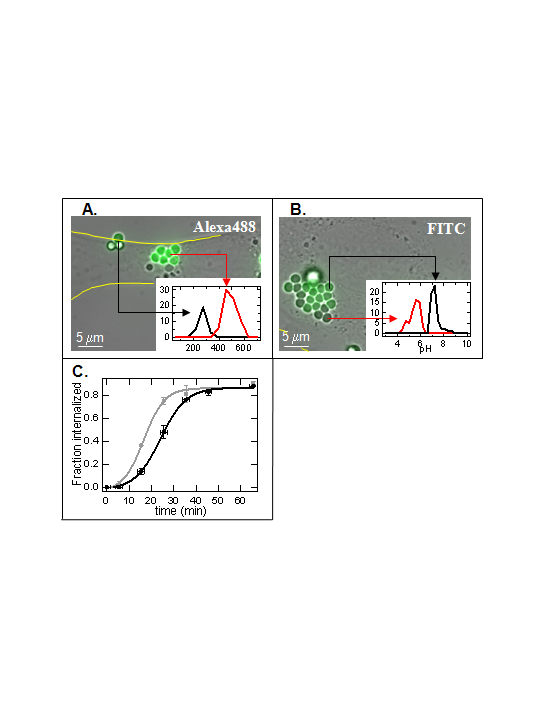

Supplement: Figure S4 — (1.35 MB TIF) [file pone.0006056.s005.tif]
